# 维莫非尼治疗BRAF^V600E^突变型Erdheim-Chester病的疗效与安全性分析

**DOI:** 10.3760/cma.j.issn.0253-2727.2021.09.007

**Published:** 2021-09

**Authors:** 婷 刘, 天骅 何, 娜 牛, 剑 李, 道斌 周, 欣欣 曹

**Affiliations:** 1 中国医学科学院、北京协和医学院北京协和医院血液内科 100730 Department of Hematology, Peking Union Medical College Hospital, Chinese Academy of Medical Sciences & Peking Union Medical College, Beijing 100730, China; 2 中国医学科学院、北京协和医学院北京协和医院核医学科 100730 Department of Nuclear Medicine, Peking Union Medical College Hospital, Chinese Academy of Medical Sciences & Peking Union Medical College, Beijing 100730, China

**Keywords:** Erdheim-Chester病, BRAF^V600E^, 维莫非尼, Erdheim-Chester disease, BRAF^V600E^, Vemurafenib

## Abstract

**目的:**

探讨维莫非尼治疗BRAF^V600E^突变型Erdheim-Chester病（ECD）的疗效和安全性。

**方法:**

回顾性分析2015年3月至2020年10月于北京协和医院接受维莫非尼治疗的12例ECD患者的临床资料、疗效、不良反应及生存情况。

**结果:**

12例患者中男7例，女5例，中位诊断年龄51.5（32～66）岁，中位受累器官数目为6（4～8）个，维莫非尼中位治疗时间为11（3～60）个月。12例患者的临床症状均得到缓解，其中完全缓解2例，部分缓解10例。7例患者在使用维莫非尼治疗前后均行^18^F-FDG-PET/CT检查，其中2例患者达到完全代谢缓解，5例达到部分代谢缓解。常见的药物不良反应为皮疹（58.3％）、关节痛（25.0％）和消化道反应（16.7％），常见不良反应事件评价标准5.0版分级均为1～2级。中位随访时间为13.5（3～60）个月，1例中枢神经系统受累患者因脑血管事件死亡，1例患者停药10个月后复发。预计2年总生存率和无进展生存率分别为88.89％和66.67％。

**结论:**

维莫非尼治疗BRAF^V600E^突变型ECD安全、有效。

## Abstract (en)

**Objective:**

To evaluate the safety and efficacy of vemurafenib in the treatment of BRAF^V600E^-mutated Erdheim-Chester disease (ECD).

**Methods:**

We retrospectively analyzed the clinical data, response rate, adverse events and survival of 12 patients with ECD treated with vemurafenib from March 2015 to October 2020 in Peking Union Medical College Hospital.

**Results:**

Of 12 patients [7 males and 5 females, median age = 51.5 (range, 32–66) years old], the median number of organs involved was 6 (range, 4–8), and the median treatment duration of vemurafenib was 11（3–60）months. All patients had improvement of clinical symptoms, of which 2 cases were completely relieved, and 10 cases were partially relieved. Seven patients evaluated by ^18^F-FDG-positron emission tomography/ computed tomography achieved a metabolic response, including 2 patients with a complete metabolic response and 5 patients with a partial metabolic response. The common adverse events in the overall cohort were grade 1 to 2 (Common Terminology Criteria for Adverse Events 5.0), including skin rash (58.3％), arthralgia (25.0％), and digestive tract reactions (16.7％). The median follow-up time was 13.5 (3–60) months. One patient with central nervous system involvement died due to a cerebrovascular event, and one patient relapsed 10 months after drug withdrawal. The estimated 2-year overall survival rate and 2-year progression free survival rate were 88.89％ and 66.67％, respectively.

**Conclusion:**

Vemurafenib is safe and effective in the treatment of BRAF^V600E^-mutated ECD.

Erdheim-Chester病（Erdheim-Chester disease, ECD）是一种罕见的非朗格汉斯细胞组织细胞疾病，50％ ～100％的ECD患者携带BRAF^V600E^突变[1]–[5]。维莫非尼是BRAF^V600E^的选择性抑制剂，通过结合BRAF^V600E^激酶的ATP位点抑制其活性，从而抑制MAPK通路（RAS-RAF-MEK-ERK）激活[6]，已被美国食品药品监督管理局（FDA）批准用于BRAF^V600E^突变型ECD患者的治疗[7]，目前国内尚无此类报道。本研究对北京协和医院12例接受维莫非尼治疗的BRAF^V600E^突变型ECD患者的临床资料进行回顾性分析，旨在探讨该药治疗ECD的疗效和安全性。

## 病例与方法

1. 病例：纳入2015年3月至2020年10月在北京协和医院确诊为BRAF^V600E^突变型并接受维莫非尼治疗的ECD患者12例。ECD的诊断基于典型的影像学表现及特征性病理学表现：①影像学表现：骨骼X线发现双侧长骨骨干对称性骨硬化或骨扫描发现特征性股骨远端和胫骨近端的放射性浓聚；②病理学：受累部位活检可见组织中存在成片泡沫状组织细胞，伴炎性细胞和多核巨细胞（Touton细胞）浸润，组织细胞表达CD68和CD163，但不表达CD1α或CD207，电镜下无Birbeck颗粒，S100可为阳性[7]–[8]。确诊ECD后再通过免疫化学染色或PCR方法筛查BRAF^V600E^突变[3]。若患者同时符合ECD及朗格汉斯细胞组织细胞增生症（LCH）诊断，则诊断为混合组织细胞疾病（ECD合并LCH）[9]。收集患者的人口学、器官受累、实验室检查、影像学检查和生存情况等数据。

2. 治疗：维莫非尼480 mg每日2次口服，治疗至疾病进展、出现无法耐受的不良反应或患者主动要求停止治疗。

3. 疗效标准：根据患者临床症状及影像学改善情况进行疗效评估。临床症状改善情况采用以下标准：完全缓解：由ECD导致的临床症状完全消失；部分缓解：由ECD导致的临床症状部分缓解；无效：临床症状无改善或恶化。

靶病变定义为治疗前^18^F-FDG-PET/CT检测到的最大标准化摄取值（SUVmax）最高的病变，参照PERCIST标准将患者分为完全代谢缓解（CMR）、部分代谢缓解（PMR）、代谢恶化（PMD）和代谢无变化（SMD）[10]。CMR：靶病灶^18^F-FDG-PET/CT摄取完全消失；PMR：靶病灶摄取值降低30％；PMD：靶病灶摄取值增加30％；SMD：非CMR、PMR和PMD。同时采用MRI或CT评估肺、心血管病变改善情况。

4. 随访：采用门诊或电话进行随访，随访时间截止至2021年2月28日。无进展生存（PFS）期定义为开始维莫非尼治疗至疾病进展、死亡或末次随访的间隔时间。总生存（OS）期定义为开始维莫非尼治疗至因任何原因死亡或末次随访的间隔时间。

5. 统计学处理：采用SPSS 21.0统计学软件进行分析，采用Kaplan-Meier法进行生存分析，*P*<0.05为差异有统计学意义。

## 结果

1. 一般临床资料：12例患者中男7例，女5例，中位诊断年龄51.5（32～66）岁，1例患者为混合组织细胞病（ECD合并LCH）。所有患者起病时均有临床症状，具体为骨痛（3例）、胸闷（3例）、多饮多尿（2例）、皮疹（2例）、突眼（2例）、腹胀（2例）、发热（2例）、肌肉包块（1例）、肢体无力（1例）。从首次出现ECD相关症状至确诊的中位间隔时间为25.5（3～133）个月。中位受累器官数目为6（4～8）个，受累器官包括：骨骼12例（100％）、肺11例（91.7％）、腹膜后10例（含肾脏，83.3％）、中枢神经系统（CNS）7例（58.3％）、心脏6例（含心包，50.0％）、血管6例（50.0％）、胰腺4例（33.3％）、垂体4例（33.3％）、皮肤3例（25.0％）、肌肉2例（16.7％）、胸膜1例（8.3％）、肠系膜1例（8.3％）、骨髓1例（8.3％）、鼻窦1例（8.％）、眶后1例（8.3％）、甲状腺1例（8.3％）、脊神经1例（8.3％），详见表1。

**表1:** 12例BRAF^V600E^突变型Erdheim-Chester病患者的临床特征、治疗情况及不良反应

例号	年龄（岁）	性别	受累器官	既往治疗	维莫非尼治疗时间（月）	临床症状改善情况	不良反应
1	51	女	骨骼、肺、腹膜后、心脏、血管、肌肉	干扰素α	20	完全缓解	皮疹
2	40	女	骨骼、肺、腹膜后、垂体、胰腺、皮肤	干扰素α	17	完全缓解	恶心、呕吐、头痛、咳嗽
3	60	男	骨骼、肺、腹膜后、心脏、血管、眼眶	干扰素α	10	部分缓解	皮疹、关节痛
4	52	男	骨骼、肺、腹膜后、心脏、血管、CNS	无	14	部分缓解	皮疹、毛发脱落
5	59	男	骨骼、肺、腹膜后、心脏、垂体、上颌窦、胰腺、肌肉	无	9	部分缓解	无
6	32	男	骨骼、肺、腹膜后、血管、垂体、胰腺	无	12	部分缓解	皮疹
7	34	女	骨骼、肺、腹膜后、心脏、胰腺、肝脏、肠系膜、CNS	干扰素α	6	部分缓解	无
8	55	男	骨骼、肺、皮肤、CNS	干扰素α	60	部分缓解	皮疹
9	45	男	骨骼、肺、腹膜后、胸膜、CNS	干扰素α	4	部分缓解	关节痛
10	66	女	骨骼、肺、腹膜后、心脏、血管、CNS、骨髓	干扰素α	15	部分缓解	皮疹
11	59	女	骨骼、肺、CNS、甲状腺	无	3	部分缓解	皮疹、胃部不适
12	50	男	骨骼、腹膜后、血管、垂体、CNS、皮肤、脊神经	干扰素α	3	部分缓解	关节痛

注：CNS：中枢神经系统

2. 治疗与疗效：12例患者中4例一线采用维莫非尼治疗；8例患者一线采用干扰素α治疗，其中6例为干扰素α治疗过程中出现病情进展后改用维莫非尼，余2例因患者意愿改用维莫非尼治疗。所有患者维莫非尼初始剂量均为480 mg，每日2次，中位治疗时间为11（3～60）个月。

12例患者的临床症状均得到缓解，其中2例（18.2％）临床症状完全缓解（皮疹、肌肉包块完全消失）；10例（81.8％）临床症状部分缓解。7例患者使用维莫非尼治疗前后均行^18^F-FDG-PET/CT检查，靶病灶末次SUVmax与基线SUVmax相比，2例（28.6％）患者达到CMR，5例（71.4％）达到PMR；SUVmax中位降低75.1％，差异有统计学意义（*P*＝0.018）。

5例肺受累患者治疗前后均行胸部CT检查，其中3例肺内病变较前好转，2例病变稳定。2例心血管受累患者治疗前后行心肌MRI及CT血管成像检查评估，右心房及大血管旁软组织病变较前明显缩小。

3. 不良反应：维莫非尼治疗过程中不良反应依次为皮疹（7例，58.3％）、关节痛（3例，25.0％）、消化道反应（2例，16.7％）、毛发脱落（1例，8.3％）、头痛（1例，8.3％）、咳嗽（1例，8.3％），根据常见不良反应事件评价标准（CTCAE）5.0版，分级为1～2级。不良反应多在治疗初期出现，随着治疗时间延长症状自行减轻并消失，无CTCAE 3级及以上不良反应发生。1例患者在服药6个月后因皮疹（CTCAE 2级）自行停服维莫非尼，停药10个月后临床症状复发，再次服用维莫非尼480 mg每日2次，皮疹未再发，临床症状再次缓解。

4. 生存分析：至末次随访时，中位随访时间13.5（3～60）个月。1例CNS受累患者在用药6个月时因脑血管事件死亡。1例患者停药10个月后症状复发，再次服用维莫非尼后症状缓解。预计2年OS率、PFS率分别为88.9％、66.7％，中位PFS和OS时间未达到（图1）。

**图1:**
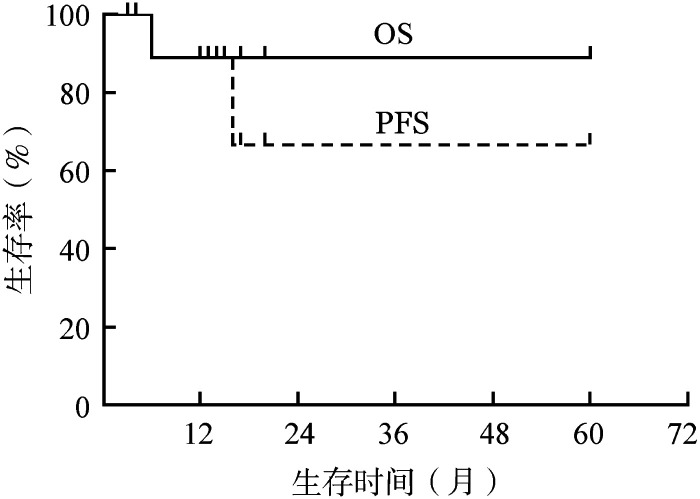
12例BRAF^V600E^突变型Erdheim-Chester病患者维莫非尼治疗后的总生存（OS）和无进展生存（PFS）曲线

## 讨论

ECD的传统治疗包括干扰素α、糖皮质激素、细胞毒性药物、IL-1受体拮抗剂、放疗和手术等[7]。干扰素α曾被推荐用于ECD患者的一线治疗，有效率为50％ ～80％[11]–[14]。但随着MAPK通路（RAS-RAF-MEK-ERK）激酶突变或重排陆续被发现[15]，靶向治疗成为了ECD患者的治疗新选择。

维莫非尼是一种BRAF抑制剂，既往用于黑色素瘤患者，可提高携带BRAF^V600E^突变的转移性黑色素瘤患者的生存率[16]。2013年，Haroche等[17]首次将维莫非尼用于3例携带BRAF^V600E^突变的多系统难治性ECD患者，3例患者均达到部分缓解。在VE-BASKET Ⅱ期临床试验中，22例BRAF^V600E^突变型ECD患者（15例既往接受过全身治疗，7例为初治患者）接受了维莫非尼960 mg每日2次治疗，所有患者病情稳定或好转，总体缓解率为54.5％（RECIST 1.1标准），FDG-PET/CT评估总体有效率达100％[18]，2年PFS率、2年OS率分别为83％、95％。基于此研究，2017年维莫非尼被美国FDA批准用于治疗BRAF^V600E^突变型ECD患者。

Haroche等[8],[19]发现，与靶向治疗相比，基于干扰素的方案治疗反应通常更为缓慢，且不同部位治疗反应不一，如骨骼和头颈部受累者治疗缓解率较低（20％～25％）[7]，心脏、CNS受累者往往需要更大的治疗剂量[20]，而多达50％的患者使用干扰素后出现难以忍受的不良反应，如发热、流感样症状、疲劳、关节痛、肌痛、抑郁等[7]。故2020年ECD国际指南推荐，对于心脏/CNS受累或伴有终末器官功能障碍的BRAF^V600E^突变型ECD患者，维莫非尼应作为一线治疗[7]；而对于无心脏/CNS或终末器官功能障碍的BRAF^V600E^突变型ECD患者，可考虑采用BRAF抑制剂或传统治疗。

本研究12例患者中，8例既往使用干扰素α治疗，4例为初治患者。所有患者临床症状均得到缓解，其中2例达到完全缓解，10例达到部分缓解；7例患者经^18^F-FDG-PET/CT评估，其中2例患者达到CMR，5例达到PMR。这些结果表明维莫非尼治疗BRAF^V600E^突变型ECD患者疗效显著。本研究中6例患者在使用干扰素α治疗过程中出现病情进展，改用维莫非尼治疗后病情缓解，故对于干扰素α耐药的BRAF^V600E^突变型ECD患者，二线治疗可考虑维莫非尼。

在VE-BASKET Ⅱ期临床试验中，大部分患者在治疗过程中因不良反应（关节痛、斑疹性皮疹、疲劳、脱发、QT间期延长、皮肤乳头状瘤和角化过度等）需将维莫非尼减量至480 mg每日2次，但减量后仍能维持疗效；1例患者因继发皮肤鳞状细胞癌停药[18]。本研究12例患者维莫非尼初始剂量均为480 mg每日2次，最常见的不良反应为皮疹（58.3％），其次为关节痛（25.0％）和消化道反应（16.7％），CTCAE分级1～2级，但多在治疗初期出现，随着治疗时间延长症状可自行缓解。其中1例患者曾因皮疹停药，但恢复服药后皮疹未再发。尚未发现维莫非尼治疗相关继发性肿瘤，患者整体对此药耐受性良好。

Cohen等[21]的一项研究纳入了51例使用维莫非尼或达拉非尼（另一种BRAF抑制剂）治疗的BRAF^V600E^突变ECD患者，75％的患者在停止BRAF抑制剂治疗后病情复发，中位复发时间为6个月，但所有患者在恢复治疗后病情均改善。而在本研究中，1例患者在服用维莫非尼6个月后因皮疹自行停药，10个月后临床症状复发，恢复服用维莫非尼后再次获得临床缓解。维莫非尼使用时间尚无定论，2020年ECD国际指南推荐维莫非尼持续使用直至疾病进展或出现不可耐受的不良反应[7]，若疾病缓解良好可考虑减量维持治疗。组织细胞疾病患者对BRAF抑制剂耐药者罕见，既往报道中仅有1例患者使用达拉非尼治疗BRAF^V600E^突变型ECD出现耐药[22]，尚无BRAF^V600E^突变型ECD患者使用维莫非尼后出现耐药的报道。

综上，维莫非尼治疗BRAF^V600E^突变型ECD患者疗效显著，患者耐受性良好，可作为BRAF^V600E^突变型ECD患者的治疗首选或难治复发患者的治疗选择。
